# Nano-Pulse Stimulation for the Treatment of Pancreatic Cancer and the Changes in Immune Profile

**DOI:** 10.3390/cancers10070217

**Published:** 2018-06-27

**Authors:** Siqi Guo, Niculina I. Burcus, James Hornef, Yu Jing, Chunqi Jiang, Richard Heller, Stephen J. Beebe

**Affiliations:** 1 Frank Reidy Research Center for Bioelectrics, Old Dominion University, Norfolk, VA 23508, USA; nburcus@odu.edu (N.I.B.); jhorn021@odu.edu (J.H.); yjing@odu.edu (Y.J.); cjiang@odu.ed (C.J.); RHeller@odu.edu (R.H.); SBeebe@odu.edu (S.J.B.); 2 Department of Electrical and Computer Engineering, Batten College of Engineering and Technology, Old Dominion University, Norfolk, VA 23508, USA

**Keywords:** nano-pulse stimulation, nanosecond pulsed electric fields, pancreatic cancer, ablation, immune response, abscopal effect, vaccine-like protection, tumor microenvironment, T regulatory cells, myeloid derived suppressor cells

## Abstract

A Pancreatic cancer is a notorious malignant neoplasm with an extremely poor prognosis. Current standard of care is rarely effective against late-stage pancreatic cancer. In this study, we assessed nanopulse stimulation (NPS) as a local treatment for pancreatic cancer in a syngeneic mouse Pan02 pancreatic cancer model and characterized corresponding changes in the immune profile. A single NPS treatment either achieved complete tumor regression or prolonged overall survival in animals with partial tumor regression. While this is very encouraging, we also explored if this local ablation effect could also result in immune stimulation, as was observed when NPS led to the induction of immune-mediated protection from a second tumor challenge in orthotopic mouse breast and rat liver cancer models. In the Pan02 model, there were insufficient abscopal effects (1/10) and vaccine-like protective effects (1/15) suggesting that NPS-induced immune mechanisms in this model were limited. To evaluate this further, the immune landscape was analyzed. The numbers of both T regulatory cells (Tregs) and myeloid derived suppressor cells (MDSCs) in blood were significantly reduced, but memory (CD44^+^) T-cells were absent. Furthermore, the numbers of Tregs and MDSCs did not reduce in spleens compared to tumor-bearing mice. Very few T-cells, but large numbers of MDSCs were present in the NPS treated tumor microenvironment (TME). The number of dendritic cells in the TME was increased and multiple activation markers were upregulated following NPS treatment. Overall, NPS treatments used here are effective for pancreatic tumor ablation, but require further optimization for induction of immunity or the need to include effective combinational NPS therapeutic strategy for pancreatic cancer.

## 1. Introduction

Though pancreatic cancer with relatively low incidence counts for a mere 2% of all cancers, with an overall 5-year survival of only 6%, it is one of most deadly cancers globally [1,2]. The incidence of pancreatic cancer has dramatically increased among people over 55, with the median age at diagnosis being 70 [3]. The incidences and mortalities are higher in the developed countries than in developing countries [1]. A trend of decreasing mortality in other major cancers has not been seen in pancreatic cancer [4]. According to the NCI SEER program [3], deaths due to pancreatic cancer have surpassed that of breast cancer and have become the 3rd leading cause of cancer death in 2017 in the USA. With global population aging and the dominance of the westernized life style [4,5] but no significant advance in the treatment and early diagnosis, pancreatic cancer will continue to be a serious global health issue.

Currently, there are no reliable screening tests or early diagnostic approaches recommended by the US Preventive Services Task Force (USPSTF) for pancreatic cancer. The majority of pancreatic cancer cases are at a late stage when diagnosed [3]. Global genomic analysis has shown there are large amounts of mutations, activation of various signaling pathways and molecular heterogeneity associated with this type of cancer [6]. A high tumor burden and biological complexity make the current management and new therapeutic approaches rarely successful, with a 5 year overall survival rate at only a single digit, in contrast to major cancers with 5 year overall survival rates that are all above 50% [3]. The Gemcitabine based or the FOLFIRINOX chemotherapy treatments can only extend the overall survival rate for a few weeks to a few months [7]. In the last decade a novel physical technology, irreversible electroporation (IRE), has emerged as a non-thermal ablation method for a number of cancer types [8,9,10] including pancreatic cancers [11,12,13,14]. A review of multiple studies suggests there is a potential prognostic benefit from IRE treatment for pancreatic cancer while more high-quality research is needed to draw a definitive conclusion [15].

Recently, another electrical engineering technology, which can generate electric pulses with pulse durations in the nanosecond ranges, has been developed by Schoenbach et al. [16] and other groups [17]. Nanosecond pulsed electric fields or electric pulses (nsPEFs or nsEPs), here named Nano-Pulse Stimulation (NPS), have been studied largely for local tumor ablation. Similar to IRE, NPS is delivered with a specialized electrode and is usually considered non-thermal if appropriate parameters are adopted. NPS successfully ablates local tumors with no reoccurrence in several animal models [18,19,20]. Beyond the local tumor regression, the protection of no tumor growth from a second tumor challenge was reported from two research groups [18,21]. Our recent data [22] demonstrated that there was not only the protective effect of tumor-free animals, but also the reduction of spontaneous distant metastases in a poorly immunogenic breast cancer model after NPS treatment. The analysis of local and systemic changes in the immune profile further suggests antitumor immunity is induced following NPS treatment.

Previously, only two studies of NPS treatment on pancreatic cancer were reported [23,24]. Because human pancreatic cancer in immune deficient animals was treated by NPS in those two studies, we were unable to evaluate whether immune responses were elicited following the NPS tumor ablation. To evaluate whether NPS ablation could induce an antitumor immune response and its potential benefit for advanced pancreatic cancer, we employed a syngeneic mouse Pan02 pancreatic cancer model to study the therapeutic efficacy of NPS for both local tumors and distant lesions in this study. Tumor-free animals were re-challenged with the same pancreatic tumor to assess if a vaccine-like protection effect occurred. Local and systemic immune profiles were characterized to gain more details on the potential antitumor immune response.

## 2. Results

### 2.1. Complete Regression or Prolonged Survival Resulted from NPS Treatment for Pancreatic Cancer

**Figure 1:**
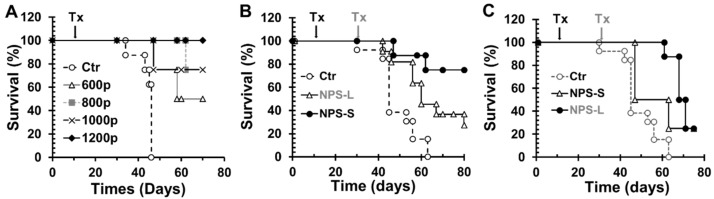
Kaplan-Meier survival curves of mice treated with NPS. Pan02 pancreatic tumors with the size of 4–7 mm (small) or 8–11 mm (large) were treated with NPS (200 ns, 30 kV/cm, 2 Hz with various pulses numbers) at day 11 or day 31 indicated by arrow. Ctr: mice with tumor but no treatment. (**A**) Dose response of NPS. 600p, 800p, 1000p or 1200p: treated with NPS with 600, 800, 1000 or 1200 pulses (*n* = 4 each group). (**B**) Different efficacy of NPS for small or large tumors treated. NPS-S: small tumors treated with NPS 800 to 1200 pulses using pinch electrodes (*n* = 8); NPS-L: large tumors treated with NPS 800 to 1200 pulses using needle electrodes (*n* = 11). (**C**) Survival curves of animals with partial regression. NPS-S (*n* = 4) or NPS-L (*n* = 8).

First we did a dose-escalating experiment to assess the safety and efficacy of NPS for the treatment of local tumor with sizes of 4–7 mm in diameter or 80–120 mm^3^ in volume. As shown in Figure 1A, NPS using a pinch electrode (200 ns, 2 Hz and 30 kV/cm) with 600, 800, 1000, or 1200 pulses achieved 50%, 75%, 75% or 100% long-term tumor free survival rates, respectively. However, the efficacy of complete regression was reduced to 27.3% (Figure 1B) when larger tumors (8–11 mm in diameter or 300–500 mm^3^ in volume) were treated with the same 200 ns NPS with 800–1200 pulses. Since the sizes of larger tumors didn’t match the available pinch electrodes, those tumors were treated with a four-needle electrode. One issue with this needle-electrode was that an irregular tumor shape often caused the exposure of needle tips beyond the tumor and into the surrounding tissues, which can cause unwanted side effects such as the risk of intestine necrosis [25]. Thus, caution should be exercised when using needle electrodes on these tumors. Nevertheless, the majority of animals with large tumors treated with a needle electrode and all animals with small tumors treated with the pinch electrodes only showed local tumor necrosis and scab formation as described in our [22] and other groups’ previous studies [23].

In additional to complete tumor regression, we also found NPS treatment prolonged overall survival rates in animals that achieved only partial tumor regression. Median survivals were 46 days for control animals, 63 days for animals with small tumors (NPS treated) and 71 days for animals with large tumors (NPS treated) (Figure 1C). The results suggest that NPS, even when complete regression is not achieved, can result in a slightly longer median survival rate when larger tumors were treated.

### 2.2. Complete Protection or Tumor Growth Inhibition in NPS Treated Tumor Free Animals Following a Re-Challenge with Pan02 Tumor Cells

**Figure 2:**
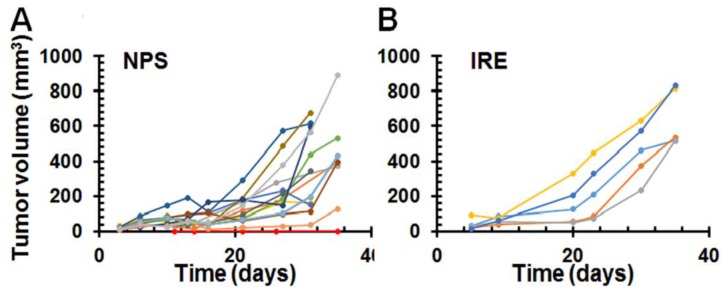
Growth curves of the second challenge tumors. Tumor-free animals after IRE or NPS treatment were challenged subcutaneously with 0.5 × 10^6^ Pan02 cells. IRE (*n* = 5): tumor-free mice after heat-assisted IRE treatment (100 µs, 1 Hz, pulse number 90 and electric fields 2100 V/cm). NPS (*n* = 15): tumor-free mice after NPS treatment using pinch electrode (200 ns, 30 kV/cm, 2 Hz with 800–1200 pulses). Each line represents a single tumor growth in one mouse.

NPS treatment has been reported to result in strong vaccine-like effects in both mouse breast cancer [22] and rat hepatocellular cancer models [18]. To assess if this also occurs in mouse pancreatic cancer, animals that were tumor-free for 7 weeks were re-challenged with 0.5 million live Pan02 tumor cells. Our results showed only 1 out of 15 mice (6.7%) treated with NPS rejected re-challenged cancer cells (Figure 2A). Noticeably, this one mouse was one of the 3 mice treated with the 100 ns NPS (50 kV/cm, 2 Hz and 800–1200 pulses), the same pulser used in the previous studies, resulting in strong protection [18,22]. In contrast, all mice treated with the 200 ns NPS (12 mice) or treated with IRE under the parameters adopted in this study (5 mice, Figure 2B) were not protected. We did notice that several re-challenge tumors grew very slowly or showed transient regression. Those growth patterns were not seen in the control animals or in the tumor-free animals following the initial IRE treatment.

### 2.3. Abscopal Effect of NPS for Existing Distant Pancreatic Tumors

**Figure 3:**
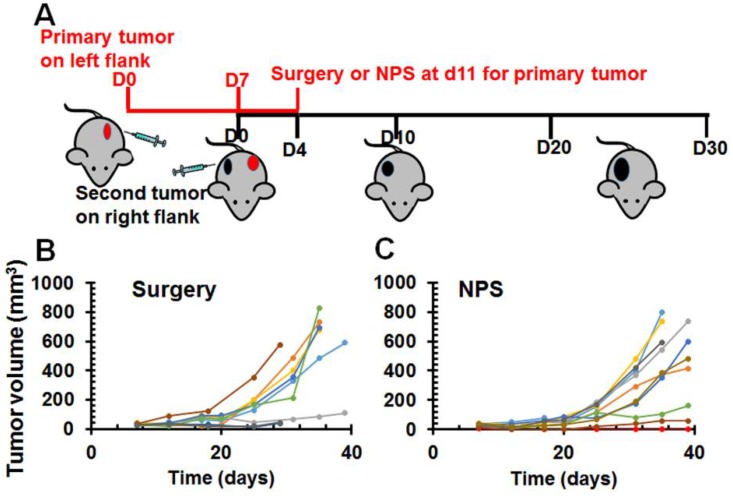
Growth inhibition of the second distant tumor lesions after primary tumor treated with NPS. (**A**) The schedule of two tumors initiated and primary one treated. (**B**) Growth curves of the second distal tumors after primary tumors were removed with surgery (*n* = 9). (**C**) Growth curves of the second distant tumors after primary tumors were treated with NPS (*n* = 10): primary tumor treated with 200 ns, 30 kV/cm, 2 Hz, 1000 pulses using pinch electrodes. Each line represents one untreated tumor growth in a single mouse.

To evaluate if local NPS tumor ablation had any effect on the distant tumor (abscopal effect), a larger and a smaller tumor were initiated on opposite flanks in each mouse. The larger one was treated with NPS (Figure 3) and the smaller one was left untreated and considered as the distant lesion. When larger tumors were ablated with NPS, 9 out of 10 smaller distant tumors grew. Only 1 mouse exhibited a protective, vaccine like effect. This was in contrast to 100% protection after NPS ablation of rat liver cancer [18] and mouse breast cancer [22]. When the larger tumors were resected, all 9 smaller distant tumors grew; none of these mice were protected from re-challenge. In this experiment, considering a potential effect of the residual tumor at primary site on immune response, a small number of animals with tumor recurrence at primary site were excluded for the evaluation of abscopal effect.

### 2.4. Various T Cell Responses after NPS Treatment for Pancreatic Cancer

**Figure 4:**
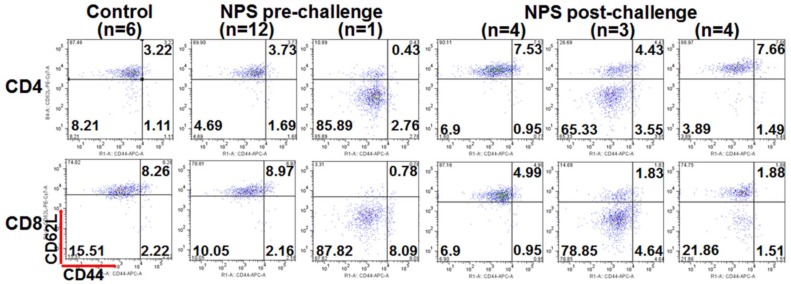
Various patterns of memory T cell responses after the second live tumor challenge. After NPS treatment mice with tumor free over 7 weeks (NPS, *n* = 13) or naïve control mice (Control, *n* = 6) were challenged subcutaneously with 0.5 × 10^6^ Pan02 cells. Blood was collected 2 days prior to challenge (pre-challenge) or 9 days after challenge (post-challenge). One representative flow cytometric graph for each pattern was shown here. (*n* = 6, 12…): numbers indicate how many animals shared the same pattern.

We previously reported that the increases of both CD4^+^ and CD8^+^ memory T cells in the tumor-free animals correlated with animal protection after treatment with NPS [22,26]. However, we did not detect the increase in the number of memory T cells including effector (CD44^+^ CD62L^−^) or central memory (CD44^+^ CD62L^+^) T cells in all tumor-free mice in the current study. In spite of this, there was a population of acute effector (CD44^−^ CD62L^−^) T cells that appeared to be induced in numerous NPS-treated mice in response to the re-challenge of live pancreatic tumor cells. After a re-challenge with Pan02 cells, 3 out of 10 mice showed a remarkable increase of acute effector T cells and 4 out of 10 mice exhibited an intermediate increase of this T cell subset (Figure 4).

**Figure 5:**
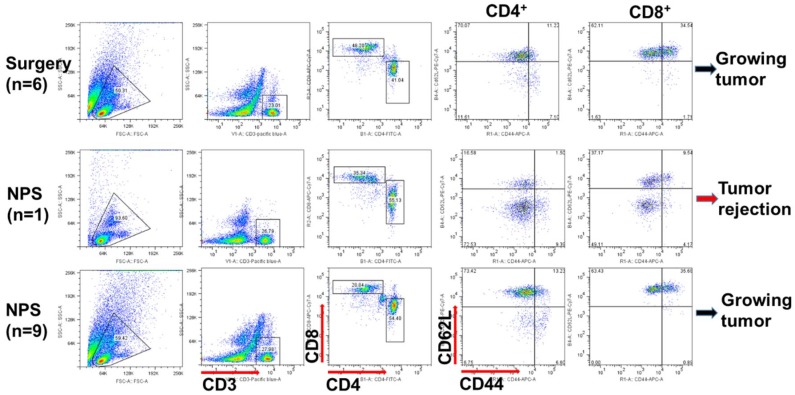
Different T cell responses after NPS treatment. In the abscopal effect experiment blood was collected 13 days after primary tumor was treated either with surgery (Surgery) or with NPS (NPS). One representative flow cytometric graph for the same pattern was shown. (*n* = 6, 1, 9): numbers indicate how many animals shared the same pattern.

Interestingly, the same pattern of T cell response was detected after NPS treatment day 13 (Figure 5). The prominent population of acute effector T cells was concomitant with the abscopal effect in the one mouse above-mentioned. But again, there was no significant effector memory T cell responses, especially for CD8^+^ effector memory T cells. Besides the above findings, we have also noticed that a prominent CD44^high^CD62L^high^ T cell subset was associated with tumor-bearing mice (Figure 5) but was not present in the tumor-free mice (Figure 4).

**Figure 6:**
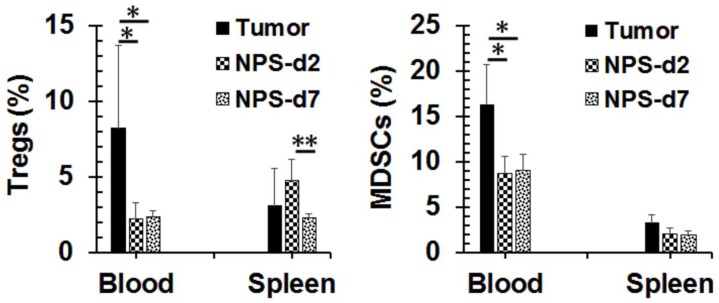
Changes of the frequencies of immune suppressor cells in the blood and spleen after NPS treatment. Blood and spleens were collected from tumor-bearing animals (tumor, *n* = 4), or animals treated with NPS on day 2 (NPS-2, *n* = 5) or day 7 (NPS-7, *n* = 5) post-treatment. Regulatory T cells (Tregs) or myeloid-derived suppressor cells (MDSCs) were analyzed with flow cytometry. * *p* < 0.05. ** *p* < 0.01 for NPS-2 or NPS-7 vs. tumor by One Way ANOVA.

Successful NPS treatment can result in a significant decrease of the peripheral blood immune suppressor cells in the 4T1 breast cancer model [22]. The same phenomenon occurred in our Pan02 tumor model (Figure 6). After NPS treatment, the number of T regulatory cells (Tregs) in peripheral blood declined from the pre-treatment level of 8.5% to the post-treatment levels of 2.3% on average. Meanwhile the number of myeloid derived suppressor cells (MDSCs) significantly reduced from 16.3% to 8.7% upon NPS treatment as well. However, these changes were not seen in the spleen.

### 2.5. Changes of Tumor Microenvironment (TME) after NPS Treatment for Pancreatic Cancer

**Figure 7:**
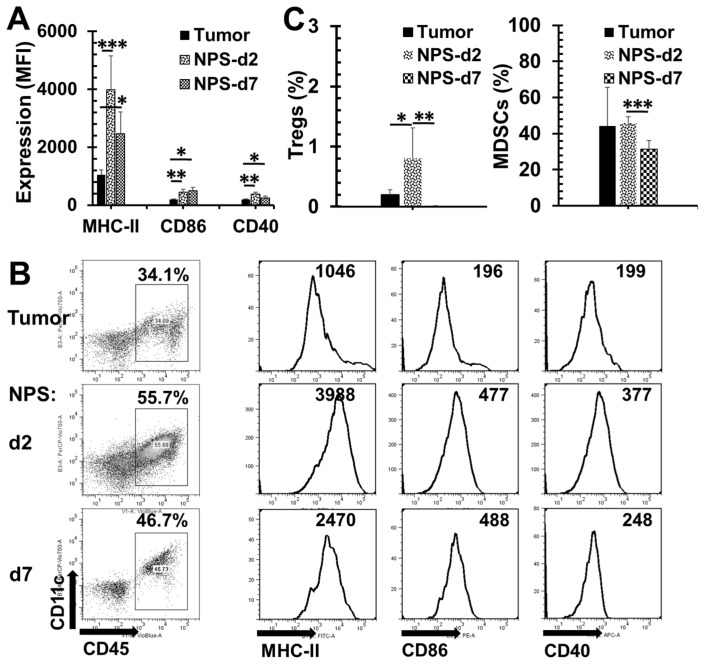
Activation of Dendritic cells (DCs) and changes of immune suppressor cells in tumor microenvironment (TME). Tumors were collected from tumor-bearing animals (tumor, *n* = 4), or animals treated with NPS on day 2 (NPS-2, *n* = 5) or day 7 (NPS-7, *n* = 5) post-treatment. Single cell suspension of tumor tissue was prepared and analyzed for the activation marker expression of DCs (**A**,**B**) and the frequencies of Tregs and MDSCs (**C**) by flow cytometry. * *p* < 0.05, ** *p* < 0.01, *** *p* < 0.001 for NPS-2 or NPS-7 vs. tumor by One Way ANOVA.

We further studied the changes of immune cell infiltration in the TME including the numbers of immune suppressor cells and the activation status of dendritic cells (DCs). We observed that the quantity of DCs was increased and all three activation biomarkers including MHC-II, CD40 and CD86 were upregulated on days 2 and 7 after NPS treatment (Figure 7A,B). However, more Tregs were present on day 2 but disappeared almost completely on day 7 post-treatment (Figure 7C). MDSC numbers were maintained at the same level on day 2 post-treatment compared to pre-treatment, but was significantly reduced on day 7 post-treatment (Figure 7C).

## 3. Discussion

Currently even the best therapeutic approaches available for pancreatic cancer are not very effective and certainly do not meet the needs of the vast majority of patients. A number of new therapeutic modalities including various ablation technologies [27] and targeted agents [28] have been developed and studied. Nevertheless, the results from these studies have been mostly disappointing or inconclusive. Ablation of locally advanced pancreatic cancer with IRE has shown survival benefit. However, the high rate of recurrence is a big issue limiting its long-term benefit [29]. Previously, a potent immune protection resulting from NPS tumor ablation was shown in both mouse breast [22] and rat hepatocellular cancers [18]. The induction of memory T cell response and remarkable diminution of spontaneous distant metastases following NPS treatment was reported by our group [22]. If the enhancement of tumor immunogenicity due to NPS treatment could be reproduced in pancreatic cancer, it would be very helpful because the majority of cases are diagnosed at advanced stages. Unfortunately, results from this study show an opposite picture when pancreatic cancer is treated by NPS with slightly different parameters from those used in the previous studies. The strong anti-tumor immunity reported previously [18,22] is not significant in this case.

The dose of NPS adopted in this study was much larger than that in the previous study in which the human xenograft pancreatic cancer was treated with 500 pulses of 100 ns NPS [23]. To achieve more than a 75% complete regression of mouse Pan02 tumors in our study, 800 to 1200 pulses were needed at 30 kV/cm, 200 ns NPS, whereas 84.2% (16/19) regression rate of Capan-1 xenograft tumors could be obtained with 30 kV/cm and 500 or 1000 pulses, 100 ns NPS. Though the reason for this discrepancy is unclear, we speculate that the large impedance of Pan02 tumor, which was reported in our previous paper [25], may require higher energy to achieve the same level of cytotoxicity. This is consistent with the observation that the higher electric field appears necessary for IRE to achieve tumor regression of Pan02 comparing to standard IRE protocol for human pancreatic cancer [30], which was reported to have much lower impedance [31].

One important question is whether NPS ablation could kill cancer stem cells or whether on the contrary it may activate cancer stem cells by the selective eradication of proliferating cells. This provocation of stem cells after pancreatic cancer ablated with IRE treatment has been suggested by Philips et al. [30]. We have also observed that the growth of recurrent tumor was accelerated after IRE treatment. However, we did not observe a significant change of growth rate in those recurrent tumors after NPS treatment. Our speculation is that NPS could kill cancer stem cells as effectively as proliferating cells. Currently, we are investigating this critical issue and will report the results when data are available.

Pancreatic cancer is known as a non-immunogenic malignancy and is notoriously resistant to most effective immunotherapeutic agents, such as immune checkpoint inhibitors [32,33]. Pan02 murine pancreatic cancer was resistant to both PD-L1 [34,35] and CTLA4 [35] antibodies. NPS induces a strong memory T cell response concomitant with the immune protection in a poorly immunogenic 4T1 breast cancer model [22]. However, NPS failed to elicit both effector and central memory T cell responses after local Pan02 tumor eradication in this study. Nevertheless, we do observe an acute effector T cell response following NPS treatment or after live tumor re-challenge. This response exhibits a low incidence rate (10–20%) after NPS treatment and the response rate is relatively higher (30%) after tumor re-challenge, but still is not sufficient to protect animals from secondary tumor growth. These data suggest there is likely a defect present in the memory T cell development after Pan02 tumors are treated with NPS.

Tregs and MDSCs are two major cell types in the immune suppressive network that promote the escape of antitumor immunity and contribute to the development of resistance to immunotherapies [36]. The numbers of both Tregs and MDSCs are elevated in the peripheral blood of tumor-bearing animals, but significantly reduced as early as 2 days after the NPS treatment. This phenomenon is likely related to the reduction of tumor burden, which has also been previously observed in the 4T1 breast cancer model [22]. Following NPS treatment, the significant diminution of Tregs and MDSCs is seen in TME as well as on day 7 post-treatment. It appears there is a spike of Treg increase in the TME as well as in the spleen on day 2 post-treatment. Though we do not know the cause of this change, it certainly does not facilitate T cell response. Noticeably, there are very few CD3^+^ T cell present in the Pan02 tumor. This is consistent with other groups’ finding [34,37] that Pan02 is a “cold” tumor with low T cell infiltration. Further analysis of TME shows that DCs are recruited into the tumor and are activated following the NPS treatment. This result supports our previous study that NPS is a potent non-drug immunogenic cell death (ICD) inducer [22] and initiates immune response in this non-immunogenic cancer. However, the above dynamic changes in the immune profiles in NPS treated pancreatic tumors, especially with the lack of T cell infiltration and an unusual spike of Tregs in the TME and in the spleen, strongly suggest some additional help is needed to break the barriers of the immune suppressive environment.

**Figure 8:**
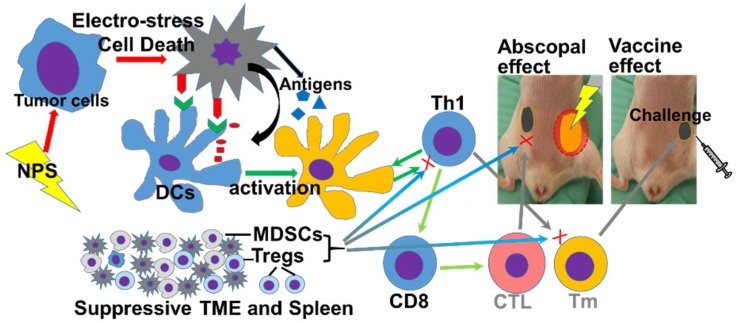
Proposed mechanisms of the failed induction of immune response with NPS treatment in the Pan02 tumor. NPS: Nano-pulse stimulation; DCs: Dendritic cells; MDSCs: Myeloid derived suppressor cells; Tregs: T regulatory cells; TME: tumor microenvironment; Th1: type I help cells. CTL: cytotoxic T cells. Tm: memory T cells.

Based on above findings, the mechanisms why NPS treatment fails to achieve a strong immune outcome in the Pan02 tumor model are proposed in the Figure 8. From Figure 7, we know NPS treatment is able to activate intratumoral DCs. However, a low rate of T cell responses (Figure 4 and Figure 5), especially a lack of both effector and central memory T cell responses (Figure 4) is directly correlated with an insufficient immune protection (Figure 2) and abscopal effect (Figure 3). Most likely, a transient increase of Tregs in the spleen (Figure 6) and tumor (Figure 7C) would block potential T cell activation, although DCs have been activated (Figure 7A,B) by the NPS treatment. In addition, a large amount of MDSCs existing at the day 2 initiation phase of immune response might be involved in this blockage effect as well. TME is believed to play a critical role in resistance to immunotherapy in pancreatic cancer [38]. This concept is supported by our above findings as well. It appears TME serves a major “brake” to the in situ vaccination with NPS treatment (Figure 8). We hypothesize that a combination therapy with NPS and an immunomodulator targeting the immunosuppressive components in TME could be more effective than the NPS treatment alone to induce strong immune responses.

We would like to point out that the parameters of NPS adopted in this study were different from those of the 100 ns NPS, the latter being employed to induce potent immune protection in the rat liver [18] and the mouse breast cancer [22] models. We did treat some tumor-bearing mice with the 100 ns NPS, but only one out of three tumor-free animals (1/3 or 33.3%) acquired the protection from the second tumor challenge. Because this is a very small number of animals, a question has been raised: Does this result actually indicate that the parameters of NPS in this study might not be optimal for the Pan02 tumor? Nevertheless, this result suggests that with the same parameters of the 100 ns NPS, the protection rate is expected to be lower in the Pan02 tumor model than the 100% protection rate observed in both the rat liver and the mouse breast cancer models [18,22].

It is clear from this current data that NPS can be effective in reducing or eliminating pancreatic tumors. It is also clear that while not effective in protecting from recurrence or inducing an abscopal effect, there were changes induced in immune cell composition. This suggests that additional studies are needed, such as molecular signals behind these findings, parameter optimization for immune outcomes, more information from different time points (we only did post-treatment days 2 and 7), etc. Currently, we are working on these important issues raised in this study. While the current results are encouraging and some encouraging discoveries were obtained, such as the potential of treatment with 100 ns NPS. That study needs to be expanded to fully evaluate that possibility. It is also clear from this work that the suppressor environment was reduced, but additional agents are needed to stimulate memory and effector T-cell responses. These will be the next approach tested to determine if combination therapies can be developed that take advantage of the effective tumor ablation and TME altering aspects of NPS while also stimulating a robust immune response.

## 4. Materials and Methods

### 4.1. Reagents and Antibodies

Carprofen (Rimadyl^®^ Injectable, 50 mg/mL) was purchased from Patterson Companies (Saint Paul, MN, USA). Pacific Blue™ anti-mouse CD3, FITC anti-mouse CD4, PerCP anti-mouse CD8a, PE anti-mouse/human CD11b, APC/Cy7 anti-mouse Ly-6G/Ly-6C (Gr-1), PE/Cy7 anti-mouse CD62L, APC anti-mouse/human CD44, APC anti-mouse CD25, Biolegend PE anti-mouse/rat/human FOXP3, PerCP anti-mouse CD11c, PE/Cy7 anti-mouse CD86, FITC anti-mouse I-A/I-E, and APC anti-mouse CD40 were purchased from Biolegend (San Diego, CA, USA). Mouse tumor dissociation kit (No. 130-096-730) was purchased from Miltenyi Biotec (Bergisch Gladbach, Germany).

### 4.2. Cell Lines

A murine pancreatic adenocarcinoma Pan02 cell line was ordered from the Division of Cancer Treatment and Diagnosis (DCTD, Bethesda, MD, USA), NCI and maintained in RPMI-1640 (ATCC^®^ 30-2001^TM^, Manassas, VA, USA) supplemented with 10% FBS (Atlantic Biological, Miami, FL, USA), 100 IU of penicillin and 100 µg/mL streptomycin. Cells were cultured in a 37 °C incubator supplied with 5% CO_2_.

### 4.3. Mice and Tumor Models

Female C57BL/6 mice (6–8 weeks of age) were purchased from the Jackson Laboratory (Bar Harbor, ME, USA). For tumor initiation, mice were injected with 1 × 10^6^ Pan02 cells in 50 μL Dulbecco’s phosphate buffered saline (DPBS) on the left flank. If it was live tumor challenge, 0.5 × 10^6^ Pan02 cells were inoculated on the right flank. The size of tumor was measured by digital calipers twice a week. Tumor volume was calculated by the formula: *V* = πab^2^/6, where (a) is the longest diameter and (b) is the shortest diameter perpendicular to (a) [39]. Mice were euthanized at the end of the follow-up period or when they met criterion described at experimental endpoints. All experimental protocols were approved by Old Dominion University Institutional Biosafety Committee (IBC) and Institutional Animal Care and Use Committee (IACUC). And all experiments were performed in accordance with relevant guidelines and regulations.

### 4.4. In Vivo NPS Treatment

For in vivo NPS tumor ablation, NPS were delivered to tumor tissue using a four-needle 5 × 7 mm electrode array [25] for tumors with the sizes of 8–11 mm, or two-plate pitch electrodes 6 mm or 8 mm diameters for tumors with the sizes of 4–7 mm (provided by Dr. Nuccitelli, Pulse Biosciences, Inc., Hayward, CA, USA). The pulse parameters were pulse duration 200 ns, frequency 2 Hz, applied electric fields 30 kV/cm and pulse number 600–1200 depending on experimental designs. This set of parameters was used in all experiments unless specifically mentioned. In some cases, tumors were treated with 100 ns NPS with 2 Hz, 50 kV/cm and 800–1200 pulses. Hair was thoroughly removed by using Nair treatment following shaving, and electrodes were covered with either K-Y jelly or ultrasound gel.

### 4.5. In Vivo IRE Treatment

In vivo IRE tumor ablation was described in our publication [25]. Briefly, tumors were treated with a four-needle electrode array with 5 × 7 mm gaps and the pulse parameters were pulse duration 100 µs, frequency 1 Hz, pulse number 90 and applied electric fields 2000 V/cm to 2500 V/cm.

### 4.6. Surgical Treatment

In the experiment for the assessment of an abscopal effect, two tumors were initiated in each mouse and only primary one was treated (Figure 3). Primary tumors were either resected or ablated by NPS treatment. For surgical treatment, a small skin incision around tumor was made and the entire tumor was removed. The skin was then closed with wound clips.

For all above in vivo tumor treatments, Carprofen (Rimadyl, 5 mg/kg) was given subcutaneously immediately before procedure and every day for 4 days to prevent/reduce pain caused by surgical wound or tumor necrosis.

### 4.7. Isolation of Splenocytes and Tumor Single Cell Suspension

These methods were described in detail in our previous publication [22]. We modified the preparation of tumor single cell suspension by using the mouse tumor dissociation kit (Miltenyi Biotech, Bergisch Gladbach, Germany) and following the protocol for dissociation of tough tumors described in the data sheet.

### 4.8. Flow Cytometric Analysis

For the analysis of immune cells in blood, a protocol of no lyse whole blood analysis (Miltenyi Biotech) was adopted to label T cells, MDSCs or Tregs. Briefly, 10 µL blood was incubated with antibody mixture for 10 min at 4 °C and washed with FACS buffer (2% FBS DPBS). For splenocytes or tumor single cell suspensions, cell surface staining was performed by incubation of 1 to 2 million in 100 µL complete media or FACS buffer with antibody mixture at room temperature for 30 min. Cells then were washed twice with 2 mL FACS buffer and re-suspended in 0.5 mL FACS buffer with 2.5% paraformaldehyde (PFA).

For PE anti-mouse/rat/human FOXP3 staining, True True-Nuclear™ Transcription Factor Buffer Set was used. Cells were first prepared by surface labeling of cells with anti-CD4 FITC, anti-CD8 PerCP or anti-CD25 APC, followed by intracellular nuclear staining using mAbs anti-Foxp3 PE after fixation and permeabilization.

Above samples were analyzed by MACSQuant Analyzer 10 (Miltenyi Biotech).

### 4.9. Statistical Analysis

All values are presented as the mean ± standard deviation (SD). Analysis of tumor volume at different time points will be completed by One Way ANOVA. Animal survival was analyzed with Kaplan–Meier Survival Analysis (LogRank test [25]). One Way ANOVA (3 or more groups) or the 2-tailed Student’s *t*-test (2 groups) were utilized to analyze the quantitative data, such as Tregs, MDSCs, DCs, DC activation markers (CD40), CD4/CD8^+^ T cells, etc. Statistical significance is assumed at *p* < 0.05. All statistical analysis including Kaplan-Meier Survival Analysis will be completed using the SigmaPlot 12.0 (Systat Software Ltd., San Jose, CA, USA).

## 5. Conclusions

NPS is a safe and effective technology for local tumor ablation if the electrode configuration matches tumor shape and size. Complete regression or partial regression with prolonged survival can be achieved in animals with syngeneic murine pancreatic cancer treated with NPS. Low rates of immune protection and abscopal effect are observed in the pancreatic cancer model. We have found that after NPS treatment circulatory Tregs and MDSCs have been significantly diminished but there is a lack of T cell responses, especially memory T cell development. NPS treatment is able to activate DCs in the TME but the lack of T cell infiltration and the presence of Tregs in the TME and in the spleen may halt consequent T cell activation and memory formation. The changes in the immune profile following NPS treatment suggest a combination with agents targeting Tregs and/or T cell recruitment/memory development would be potentially more effective to achieve meaningful immune outcomes.
